# The Relationship between Beverages Consumption and Cognitive Impairment in Middle-Aged and Elderly Chinese Population

**DOI:** 10.3390/nu15102309

**Published:** 2023-05-15

**Authors:** Xinting Jiang, Liang Cui, Lin Huang, Yihan Guo, Gaozhong Huang, Qihao Guo

**Affiliations:** 1 Department of Gerontology, Shanghai Sixth People’s Hospital Affiliated to Shanghai Jiao Tong University School of Medicine, Shanghai 200233, China; jiangxinting0914@163.com (X.J.); cuiliangsh@163.com (L.C.);; 2 Department of VIP Clinical, Shanghai Sixth People’s Hospital Affiliated to Shanghai Jiao Tong University School of Medicine, Shanghai 200233, China; 3 Faculty of Medicine, The University of Queensland, Brisbane 4072, Australia

**Keywords:** cognitive impairment, water, tea, coffee, pure milk, amyloid beta

## Abstract

Some evidence shows that beverage consumption has an impact on cognitive performance. This is a follow-up study of dietary habits and cognitive function in the Chinese middle-aged and elderly population. The objective of this study was to explore the relationship between beverage consumption and cognitive impairment. The source and grouping of the participants can be seen in the previous article, “Study of Diet Habits and Cognitive Function in the Chinese Middle-Aged and Elderly Population: The Association between Folic Acid, B Vitamins, Vitamin D, Coenzyme Q10 Supplementation and Cognitive Ability”. Among 892 participants, one-third (296) completed both Amyloid beta(Aβ)-PET and plasma biomarkers. The results showed that the consumption of beverages (green tea, coffee, pure milk) was a protective factor for cognitive impairment, daily water consumption <1500 mL (especially <500 mL) was a risk factor for cognitive impairment, and the above correlated with baseline cognitive status. The relationship of green tea, coffee, and pure milk consumption with cognitive impairment was related to gender. We also found that among the participants with Aβ deposition, the consumption of pure milk and green tea was associated with low levels of p-Tau-181. In conclusion, the relationship between beverage consumption and cognitive impairment in Chinese middle-aged and elderly adults may be related to baseline cognitive status, gender, and Aβ deposition.

## 1. Introduction

China has the largest number of people with dementia, accounting for nearly 25% of the world’s dementia cases and increasing at a rate of over 360,000 per year [1]. Identifying, and subsequently managing risk, is critical to slowing the progression of Alzheimer’s disease (AD)-related cognitive impairment. One modifiable factor in dementia is diet, and healthy eating habits are associated with a reduced risk of dementia [2]. Dietary intervention is essential to prevent age-related cognitive decline [3].

Adequate water intake is required to maintain the hydrated state, which benefits human health [4]. Dehydration or other hydration imbalances can lead to the dysfunction of physical or cognitive activities [4]. A German study suggested that adequate water intake improves short-term memory [5]. Despite the body’s water balance mechanism, even minor systemic water loss can impair cognitive performance [6]. However, a systematic review concluded that underhydration might not significantly impair cognitive function [7].

The relationship between tea, and coffee consumption and cognitive performance has been inconsistent in the results of previous clinical trials. A study found that green tea intake may reduce the risk of dementia and improve cognitive performance [8]. However, another study found a cognitive protective effect of black tea intake in older Chinese adults but did not find a correlation between green tea and cognitive function [9]. A 21-year longitudinal study reported that coffee consumption in middle age was associated with a reduced risk of AD/dementia in later life, and no association was found between tea consumption and AD/dementia [10]; the possible reason for this result is that coffee drinking is more common in the Finnish population and tea drinking is relatively uncommon. Several studies have also denied a significant cognitive effect of habitual coffee [10,11,12] or tea [12] consumption.

The results of two retrospective meta-analyses suggested that higher milk intake may be associated with better cognitive performance [13,14], but this may be limited to Asian populations [14]. The current evidence on the relationship between milk intake and cognitive performance is inconsistent, and no firm conclusions can be drawn about their relationship [15,16].

The relationship between water, tea, coffee, and pure milk consumption and cognitive impairment remains to be determined. Previous studies have found that the effects of beverage consumption on chronic diseases such as hypertension and Parkinson’s differ across gender groups [17,18], and there may also be differences in beverage preferences across gender groups [19]. At present, there are few studies on the relationship between beverages consumption and cognitive impairment in different gender groups.

Amyloid beta (Aβ) deposition increases the risk of cognitive decline [20]. Compared with cerebrospinal fluid biomarkers, blood-based biomarkers are safe, minimally invasive, less resource-intensive, and widely available. Compared to normal cognitive individuals, individuals with cognitive impairment had lower plasma Aβ42/40 levels [21], higher plasma neurofilament light chain (NfL) levels [22], and lower plasma tau phosphorylated at threonine 181 (p-Tau-181) [23]. In addition, it was found that the increase of plasma p-tau-181 was related to extensive cortical Aβ lesions, helping to identify people at high risk of AD [23,24]. Plasma biomarkers can not only assist in diagnosing AD but can also be used clinically to monitor the effects of lifestyle factors such as diet on cognition in people at greater risk of cognitive impairment through regular plasma biomarker testing [25]. However, few studies have paid attention to the relationship between beverage consumption and plasma biomarkers in Aβ-PET-positive populations.

The purpose of this study was to assess the relationship between water, tea, coffee, and pure milk consumption and cognitive status in middle-aged and elderly populations of different genders. We also explored the relationship between different consumptions of water, tea, pure milk, and coffee and plasma biomarkers in participants with Aβ-PET-positive(+) and Aβ-PET-negative(−) genotypes.

## 2. Materials and Methods

### 2.1. Demographic Characteristics

The recruitment methods, inclusion and exclusion criteria, and ethical review of the participants can be found in the previous article [26].

### 2.2. Data Collection

The information content of the participants can be found in the previous article. However, in this study, information on beverage (water, tea, coffee, pure milk) consumption was collected [26].

### 2.3. F-Florbetapir PET Imaging and Semi-Quantitative Analysis of PET Images

First, 18F-florbetapir was prepared by the PET center of Huashan Hospital, Fudan University. After resting for 15 min in a quiet environment, the participants received an intravenous injection of 7.4 MBq/kg 18F-florbetapir according to body weight, and PET/CT imaging of the brain was performed 50 min later using a PET/CT scanner (Biograph mCT Flow PET/CT, Siemens, Erlangen, Germany) for 20 min. The PET data were reconstructed using a filtered inverse projection algorithm. Individual PET images were rigidly fused to MRI T1 images with six parameters and spatially normalized. The PET image interpretation was performed independently by three PET diagnostics experts, and the results depended on the agreement of more than two experts.

Aβ-PET semi-quantitative analysis was performed to assess the intracerebral Aβ deposition load. Using the cerebellar peduncle as the reference area for Aβ-PET semi-quantitative analysis, the standardized uptake value ratios (SUVR) of Aβ deposition in eight gray matter cortical brain regions of interest (frontal, lateral parietal, lateral temporal, medial temporal, occipital, basal ganglia, posterior cingulate gyrus, precuneus) were defined based on automatic anatomical localization mapping (a higher SUVR value represents a heavier load of Aβ deposition in the brain) [27].

### 2.4. Measurements of Plasma Biomarkers Related to AD

Venous whole-blood samples were centrifuged at 3000 rpm and 4 °C for 10 min. Then, the upper plasma layer was aspirated and transferred to EP tubes. Plasma samples were stored in a −80 °C refrigerator after collection and transported on a −20 °C dry ice-cold chain before testing. AD-related blood biomarkers included Aβ42, Aβ40, t-Tau, p-Tau-181, and NfL. Participants were tested for AD-related blood biomarkers using the Quanterix SIMOA HD-1 digital single-molecule immunoarray analyzer. Plasma Aβ42, Aβ40, t-Tau were measured using the Neurology 3-Plex A Assay Kit (Lot 502838); plasma p-Tau-181 was measured using the p-Tau-181 Assay Kit V2 (Lot 502923); and plasma NfL was measured using the NF-light Assay Kit (Lot 202700). Pretreatment and sample loading were performed according to the manufacturer’s instructions [28]. Plasma samples were diluted 1:4 according to the minimum required dilution (MRD), with a dilution sample size of 152 µL for each measurement of Aβ42, Aβ40, t-Tau, NfL, and 100 µL for p-Tau-181 plasma samples to be tested. Magnetic beads coated with antibodies to the target, biotinylated streptavidin-β galactosidase (SBG), and β-D-galactopyranoside (RGP) were loaded on the plate in sequence. Finally, the concentrations (pg/mL) of AD-related plasma biomarkers detected in the samples were obtained based on the standard curve information.

### 2.5. Assessments

The assessment of cognitive function of the participants and the way participants were grouped can be found in the previous article [26]. However, in this study, we divided the participants into objective cognitive unimpaired and objective cognitive impairment groups according to the degree of cognitive impairment. Participants in the normal control (NC) and subjective cognitive decline (SCD) groups were included in the objective cognitive unimpaired group. Participants in the mild cognitive impairment (MCI) and AD groups were included in the objective cognitive impairment group.

The ε3/ε4 and ε4/ε4 genotypes carried the APOE ε4 allele, and the ε2/ε2, ε2/ε3, ε2/ε4, and ε3/ε3 genotypes did not carry the APOE ε4 allele [29]. Of the 892 participants, 1/3 (296) completed both Aβ-PET and plasma biomarkers. Subjects were divided into Aβ-PET (+) and Aβ-PET (−) groups based on the results of Aβ-PET.

### 2.6. Data Analysis

The method of data analysis can be found in the previous article [26]. However, in this study, we considered beverage consumption (water, tea, coffee, pure milk) as a category in the multivariate analysis.

Furthermore, the relationships between the consumption of each beverage (water, tea, coffee, pure milk) and plasma AD biomarkers were compared in the Aβ-PET (+) group using an independent sample *t*-test or one-way ANOVA (determined by the number of variables). This statistical analysis was also performed in the Aβ-PET (−) group.

## 3. Results

A comparison of participants’ demographic data, basic physical condition, drug history, smoking habits, and drinking habits and the analysis of the results can be found in the previous article [26]. 

Table 1 shows the results of the univariate analysis. The SCD, AD, and objective cognitive unimpaired groups had more APOEε4 allele carriers compared with the NC, MCI, and objective cognitive impairment groups. Compared to the NC group, the SCD group consumed coffee infrequently. Compared with the MCI group, the AD group had less daily water consumption, consumed green tea or pure milk infrequently, and had less weekly milk intake. The objective cognitive impairment group had less daily water consumption and consumed less coffee, green tea, or yogurt compared to the objective cognitive unimpaired group. The “daily milk intake” and “frequency of coffee consumption” items were not found to be associated with the severity of cognitive impairment. 

Table 2 shows the results of the multivariate analysis. Compared with those with infrequent coffee consumption, females in NC group had a lower risk of SCD from regular coffee consumption (OR = 0.500, 95%CI, 0.305–0.820, *p* = 0.006). This relationship remained statistically significant after adjusting for education, age, diarrhea, and pro-cognitive drugs use (OR = 0.479, 95%CI, 0.287–0.801, *p* = 0.005). After further adjustment for daily water consumption, long-term drinking water, pure milk consumption, tea consumption, tea drinking frequency, yogurt consumption, and Apolipoprotein E genotype in Model c, the results remained unchanged (OR = 0.459, 95%CI, 0.248–0.851, *p* = 0.013).

Table 3 shows the results of multivariate analysis. Compared with those drinking >1500 mL per day, individuals with MCI who drank 1000–1500 mL, 500–1000 mL, and <500 mL per day had a higher risk of AD (OR = 1.998, 95%CI, 1.119–3.568, *p* = 0.019; OR = 2.283, 95%CI, 1.317–3.959, *p* = 0.003; OR = 4.791, 95%CI, 2.295–10.005, *p* = 1.567, respectively). This relationship remained statistically significant after adjusting for education, age, dieting and weight loss, diarrhea, allergic history, and pro-cognitive drugs use in Model b (OR = 2.083, 95%CI, 1.076–4.032, *p* = 0.029; OR = 2.015, 95%CI, 1.071–3.792, *p* = 0.030; OR = 3.911, 95%CI, 1.721–8.887, *p* = 0.001, respectively). After further adjustment for long-term drinking water, tea consumption, tea frequency, pure milk consumption, yogurt consumption, and APOE genotype in Model c, individuals with MCI who drank <500 mL per day had a higher risk of developing AD (OR = 3.387, 95%CI, 1.447–7.927, *p* = 0.005).

Compared to those with infrequent tea consumption, females with MCI had a lower risk of AD from regular tea consumption (OR = 0.322, 95%CI, 0.177–0.585, *p* < 0.001). This relationship remained statistically significant in Model b (OR = 0.379, 95%CI, 0.197–0.729, *p* = 0.004). After further adjustment of daily water consumption, long-term drinking water, coffee consumption, pure milk consumption, yogurt consumption, and APOE genotype in Model d, the results remained unchanged (OR = 0.430, 95%CI, 0.195–0.948, *p* = 0.037).

Compared to those with infrequent pure milk consumption, females with MCI had a lower risk of AD from regular pure milk consumption (OR = 0.392, 95%CI, 0.339–0.644, *p* = 0.000). This relationship remained statistically significant in Model b (OR = 0.410, 95%CI, 0.234–0.718, *p* = 0.002). After further adjustment of daily water consumption, long-term drinking water, tea consumption, tea frequency, coffee consumption, yogurt consumption, and APOE genotype in Model e, the results remained unchanged (OR = 0.441, 95%CI, 0.231–0.843, *p* = 0.013).

Table 4 shows the results of multivariate analysis. Compared to those with infrequent tea consumption, males with objective cognitive unimpaired had a lower risk of objective cognitive impairment from regular tea consumption (OR = 0.314, 95%CI, 0.185–0.533, *p* < 0.001). This relationship remained statistically significant after adjusting for education, age, marriage, allergic history, and pro-cognitive drugs use in Model b (OR = 0.294, 95%CI, 0.161–0.537, *p* < 0.001). After further adjustment of daily water consumption, coffee consumption, pure milk consumption, yogurt consumption, and APOE genotype in Model c, the results remained unchanged (OR = 0.344, 95%CI, 0.182–0.647, *p* = 0.016).

Compared to those who basically did not drink tea per day, males with objective cognitive unimpaired with tea frequency ≥3 cups/day, 1–2 cups/day, and <1 cup/day had a lower risk of objective cognitive impairment (OR = 0.367, 95%CI, 0.201–0.671, *p* = 0.001; OR = 0.419, 95%CI, 0.216–0.812, *p* = 0.010; OR = 0.331, 95%CI, 0.141–0.770, *p* = 0.010, respectively). This relationship remained statistically significant in Model b (OR = 0.296, 95%CI, 0.146–0.692, *p* = 0.001; OR = 0.374, 95%CI, 0.176–0.792, *p* = 0.010; OR = 0.245, 95%CI, 0.090–0.669, *p* = 0.006, respectively). After further adjustment in Model c, the results remained unchanged (OR = 0.336, 95%CI, 0.174–0.772, *p* = 0.008; OR = 0.442, 95%CI, 0.201–0.974, *p* = 0.043; OR = 0.261, 95%CI, 0.093–0.733, *p* = 0.011, respectively).

Table 5 shows that in the PET (+) group, pure milk or green tea consumption (compared to none) was statistically associated with lower p-Tau-181 levels (*p* = 0.042, *p* = 0.014, respectively).

Table 6 shows that coffee or daily water consumption were statistically associated with AD-related biomarkers. 

## 4. Discussion

This study examined the intrinsic relationship between beverage consumption and cognitive abilities. The results suggest that the relationship between water, tea, coffee, and pure milk consumption and cognitive performance in middle-aged and elderly adults is related to basic cognitive status, gender, and Aβ deposition.

Water has many roles in the human body. Water in the body is the solvent that carries nutrients, the reactants and products of metabolic processes, and the main component of cells and tissues [30]. Therefore, optimal hydration is essential to maintain a range of normal physiological functions required for the health of the body [31]. A Chinese randomized controlled trial of youth water drinking reported that water restriction for 36 h impaired situational memory and mood [32]. Compared to the young population, the older population has a greater risk of dehydration due to a more inadequate water balance regulation mechanism [6]. A study found that human hydration status and water intake follow a U-shaped curve with cognition and that dehydration or overhydration can impair cognition in older adults [33]. Available data suggest that good hydration is associated with better results on cognitive tests and that mild dehydration impairs cognitive performance [34]. Our study found that daily water consumption <1500 mL (especially <500 mL) was a risk factor for middle-aged and elderly people whose cognitive abilities have been impaired. Our study provided further support for the cognitive impairment of insufficient water consumption in the middle-aged and elderly, especially those with pre-existing cognitive impairment. In contrast to our results, a study [35] found no significant relationship between hydration status and cognitive function. A possible reason for this result is that the study was conducted in normal cognitive older adults and defined inadequate water consumption in older adults as less than the appropriate intake (AI) (2000 mL for females and 2500 mL for males).

Our study found that for objective cognitive unimpaired males and females with MCI, green tea consumption was a protective factor for cognitive impairment. The mechanism was that green tea consumption improved AD pathology in participants with Aβ deposition. A Japanese meta-analysis found that green tea consumption reduced all-cause mortality in middle-aged and older adults, but there were differences between males and females [36]. A longitudinal study reported a 28% or greater reduction in the risk of cognitive decline among older adults with higher green tea consumption [37]. Another longitudinal study found that tea intake slowed the rate of cognitive decline in females, but it did not find an effect of tea consumption on cognitive performance in males [38]. It is hypothesized that there is variability in the effects of green tea intake on the cognitive profiles of gender-specific groups. This variability may be due to the gender-dependent effects of epigallocatechin gallate (EGCG) on the metabolic regulation of the organism [39]. Tea drinking originated in Asia and is particularly revered by the Chinese. Tea is usually made from the leaves of the tea tree, an economic plant, and is one of the most popular beverages due to its satisfying sensory experience and many health-promoting properties [40]. The main polyphenol present in green tea is EGCG, and previous studies have found that the improvement in cognitive performance associated with green tea intake may be the result of the combined effects of EGCG, caffeine, L-theanine, and flavonoids [8,41,42,43]. These active ingredients prevent and modulate AD pathology through multiple mechanisms, including the reduction of Aβ production, aggregation and inhibition of tau aggregation, and hyperphosphorylation [42,43]. An experimental animal study revealed that EGCG and flavonoids reduced Aβ-induced mitochondrial dysfunction in AD transgenic mice [44]. A systematic review reported that caffeine and L-theanine, alone or in combination, affected various neurotransmitter systems, improving attention, cognition, and mood [45].

Green tea has a higher concentration of polyphenols and a higher antioxidant potential than black tea [46]. Black tea is made by promoting the enzymatic oxidation of fresh leaves. Most catechins are converted to oxidized forms called theaflavins and theobromine [43]. The total catechin levels are reduced from 35−50% in green tea to 10% in black tea [43]. Meta-analyses results have suggested that the cognitive benefits of tea (green, black) may increase with increasing daily consumption [47,48,49]. Another study [9] reported a cognitive protective effect of black tea consumption. Our study did not find a correlation between black tea consumption and cognitive impairment in middle-aged and older adults. We speculate that this result may be due to the small sample of study subjects who filled in the black tea consumption or the limited extent of the effect of black tea on cognitive impairment.

Caffeine is the most widely consumed mental stimulant in the world. Our study found that for middle-aged and older females with NC, coffee consumption was a protective factor for cognitive impairment. Previous studies have found that coffee consumption improves cognitive health [47,50,51]. The 2011–2014 National Health and Nutrition Survey conducted by XueDong et al. reported that coffee and caffeine were associated with better cognitive performance [50]. MRI parameter analysis found that moderate coffee consumption (two cups per day) was associated with better white matter preservation and cerebral blood flow, especially in the elderly with normal cognitive function [52]. Jee Wook Kim et al. [53] found that coffee intake can reduce the risk of AD or related cognitive decline in older subjects with normal cognition. Marilyn et al. [54] found that women with higher levels of coffee intake scored higher on cognitive tests [54]. A clinical study found that caffeine had a more significant neuroprotective effect on women with normal cognition [55]. That is, the neuroprotective effect of coffee may be related to gender [54,55] and cognitive status [52,53,55]. Our study did not find that coffee reduced AD pathological changes in participants with Aβ deposition. However, previous studies have found that the cognitive protective effect of coffee may be associated with reduced Aβ deposition in the brain [53]. Various types of coffee, including roasted coffee and instant coffee, can reduce Aβ levels in the body, and the mechanism may be to reduce the production of Aβ [56,57]. Notably, the effect of coffee in inhibiting Aβ and tau aggregation and providing neuroprotection is not only dependent on caffeine but is also associated with Phenylindanes [58].

Our study found that for middle-aged and older females with MCI, pure milk consumption was a protective factor for cognitive impairment. For participants with Aβ deposition, the regular consumption of pure milk may improve AD pathology. Similar to our results, a prospective cohort study in a Japanese older population suggested that higher milk and dairy consumption may reduce the risk of dementia in older adults [59]. A Spanish study found that whole milk consumption was associated with higher MMSE scores in people at high cardiovascular risk [60]. However, other studies [61,62,63] have concluded that milk consumption is detrimental to cognitive function in older populations. A study found that higher milk and dairy consumption among older adults over 60 was negatively related to cognitive function, although protein consumption was positively related to cognitive performance [61]. Maija et al. [62] found that whole milk may cause cognitive decline. A prospective cohort study in France found that eating more than the recommended amount of dairy products may damage the cognitive function of older women [63]. A precise conclusion has not been reached regarding the relationship between milk intake and cognition, which may be influenced by the fat content and intake of milk. The protective effect of pure milk on cognitive impairment in middle-aged and older adults may be attributed to its content of protein, minerals, vitamins, and essential amino acids. Metabolic diseases such as type 2 diabetes, hypertension, dyslipidemia, and obesity are associated with an increased risk of cognitive impairment [3]. Milk consumption can promote cognitive function in middle-aged and elderly adults by improving neurovascular dysfunction, as well as reducing weight and the risk of metabolic diseases [64,65].

The limitation of this study can be found in the previous article [26]. The study was conducted among a specific population of middle-aged and elderly adults in Chinese, and the results may not be generalizable to other populations or age groups.

## 5. Conclusions

We found that the relationship between beverage consumption and cognitive impairment in Chinese middle-aged and elderly adults may be related to baseline cognitive status, gender, and Aβ deposition.

Daily water consumption <1500 mL (especially <500 mL) was a risk factor for cognitive impairment in those with MCI; for objective cognitive unimpaired males, tea (especially green tea) consumption was a protective factor for cognitive impairment; for females with NC, coffee consumption was a protective factor for cognitive impairment; and for females with MCI, green tea and pure milk consumption was a protective factor for cognitive impairment. We also found that in participants with Aβ deposition, the regular consumption of pure milk or green tea may improve AD pathology. Neuroimaging studies may help to further explain the mechanism underlying the neuroprotective effects of drinking water, coffee, tea, and pure milk. Our study is a cross-sectional study; therefore, prospective studies are still needed for further validation. In the future, this research field is expected to bring new approaches for preventing dementia and AD.

## Figures and Tables

**Table 1:** Description of the sample based on cognitive status.

	NC Group	SCD Group	*p* ^a^	MCI Group	AD Group	*p* ^a^	Objective Cognitive Unimpaired Group	Objective Cognitive Impairment Group	*p* ^a^
N	185	227		296	184		412	480	
APOE ε4 allele carries			0.043			<0.001			<0.001
YES	33 (18.0)	42 (18.8)		70 (23.6)	93 (50.8)		75 (18.3)	163 (34.0)	
NO	150 (82.0)	181 (81.2)		226 (76.4)	90 (49.2)		334 (81.7)	316 (66.0)	
Beverages consumption									
Daily water consumption									
>1500 mL	43 (23.2)	50 (22.4)	0.585	76 (26.0)	23 (12.8)	<0.001	93 (22.8)	99 (21.0)	0.009
1000–1500 mL	70 (37.8)	83 (37.2)		86 (29.5)	52 (28.9)		153 (37.5)	138 (29.2)	
500–1000 mL	64 (34.6)	73 (32.7)		110 (37.7)	76 (42.2)		137 (33.6)	186 (39.4)	
<500 mL	8 (4.3)	17 (7.6)		20 (6.8)	29 (16.1)		25 (6.1)	49 (10.4)	
Long-term drinking water									
Mineral water	16 (8.6)	15 (6.7)	0.585	16 (5.5)	9 (4.9)	0.024	31 (7.6)	25 (5.3)	0.007
Tea	57 (30.8)	59 (26.3)		72 (24.6)	24 (13.2)		116 (28.4)	96 (20.2)	
boiled water	92 (49.7)	125 (55.8)		174 (59.4)	128 (70.3)		217 (53.1)	302 (63.6)	
purified water	20 (10.8)	25 (11.2)		31 (10.6)	21 (11.5)		45 (11.0)	52 (10.9)	
Tea drinking frequency									
≥3 cups/day	55 (29.9)	52 (23.2)	0.120	71 (24.1)	31 (17.1)	<0.001	107 (26.2)	102 (21.5)	<0.001
1–2 cups/day	45 (24.5)	44 (19.6)		53 (18.0)	19 (10.5)		89 (21.8)	72 (15.2)	
<1 cups/day	24 (13.0)	31 (13.8)		36 (12.2)	15 (8.3)		55 (13.5)	51 (10.7)	
Rarely	60 (32.6)	97 (43.3)		134 (45.6)	116 (64.1)		157 (38.5)	250 (52.6)	
Coffee drinking frequency									
1–2 cups/WKD	42 (49.4)	38 (48.1)	0.550	53 (59.6)	20 (52.6)	0.559	80 (48.8)	73 (57.5)	0.188
3–6 cups/WKD	12 (14.1)	16 (20.3)		14 (15.7)	9 (23.7)		28 (17.1)	23 (18.1)	
Everyday	31 (36.5)	25 (31.6)		22 (24.7)	9 (23.7)		56 (34.1)	31 (24.4)	
Coffee consumption									
YES	75 (40.5)	65 (28.9)	0.013	72 (24.4)	32 (17.5)	0.075	140 (34.1)	104 (21.8)	<0.001
NO	110 (59.5)	160 (71.1)		223 (75.6)	151 (82.5)		270 (65.9)	374 (78.2)	
Tea consumption									
Green tea	58 (31.7)	63 (28.1)	0.071	88 (29.9)	34 (18.6)	0.001	121 (29.7)	122 (25.6)	<0.001
Black tea	51 (27.9)	46 (20.5)		45 (15.3)	18 (9.8)		97 (23.8)	63 (13.2)	
None	74 (40.4)	115 (51.3)		161 (54.8)	131 (71.6)		189 (46.4)	292 (61.2)	
Weekly milk intake									
Everyday	96 (52.2)	118 (52.4)	0.854	142 (48.5)	78 (43.3)	0.027	214 (52.3)	220 (46.5)	0.129
≥3 times	41 (22.3)	53 (23.6)		74 (25.3)	32 (17.8)		94 (23.0)	106 (22.4)	
<3 times	21 (11.4)	20 (8.9)		37 (12.6)	32 (17.8)		41 (10.0)	69 (14.6)	
None	26 (14.1)	34 (15.1)		40 (13.7)	38 (21.1)		60 (14.7)	78 (16.5)	
Daily milk intake									
One cup	116 (65.5)	140 (64.5)	0.992	171 (62.2)	99 (56.9)	0.112	256 (65.0)	270 (60.1)	0.458
Half a cup	28 (15.8)	35 (16.1)		52 (18.9)	31 (17.8)		63 (16.0)	83 (18.5)	
<Half a cup	11 (6.2)	13 (6.0)		18 (6.5)	8 (4.6)		24 (6.1)	26 (5.8)	
Rarely	22 (12.4)	29 (13.4)		34 (12.4)	36 (20.7)		51 (12.9)	70 (15.6)	
Yogurt consumption									
YES	114 (61.6)	139 (62.1)	0.929	161 (54.6)	87 (47.5)	0.135	253 (61.9)	248 (51.9)	0.003
NO	71 (38.4)	85 (37.9)		134 (45.4)	96 (52.5)		156 (38.1)	230 (48.1)	
Pure milk consumption									
YES	121 (65.4)	162 (72.3)	0.132	205 (69.5)	104 (57.1)	0.006	283 (69.2)	309 (64.8)	0.164
NO	64 (34.6)	62 (27.7)		90 (30.5)	78 (42.9)		126 (30.8)	168 (35.2)	

Notes: ^a^ Using the chi-square test or Fisher exact test (2-tailed). *p*, *p*-value. NC, normal control; SCD, subjective cognitive decline; MCI, mild cognitive impairment; AD, Alzheimer’s disease; WKD, Weekend. One cup = 250 mL.

**Table 2:** Multivariate analysis of coffee consumption.

Group	Categorical Variable	Model	Reference Group		B	SE	*p*	OR	95%CI
The NC group and the SCD group (female)									
	Coffee consumption								
		a	NO	YES	−0.693	0.253	0.006	0.500	0.305–0.820
		b	NO	YES	−0.735	0.262	0.005	0.479	0.287–0.801
		c	NO	YES	−0.779	0.315	0.013	0.459	0.248–0.851

Notes: a, basic model, no adjustment; b, adjusted for education, age, diarrhea, pro-cognitive drug use. c, adjusted for all variables in model b + daily water consumption, long-term drinking water, pure milk consumption, tea consumption, tea drinking frequency, yogurt consumption, Apolipoprotein E genotype. NC, normal control; SCD, subjective cognitive decline; B, Coefficient for the constant; SE, Standard error around the coefficient for the constant; *p*, *p*-value; OR, Exp (B); 95%CI, Confidence interval for the odds ratio with its upper and lower limits.

**Table 3:** Multivariate analysis of daily water consumption, tea consumption, and pure milk consumption.

Group	Categorical Variable	Model	Reference Group		B	SE	*p*	OR	95%CI
The MCI group and the AD group									
	Daily water consumption								
		a	>1500 mL	1000–1500 mL	0.692	0.296	0.019	1.998	1.119–3.568
			>1500 mL	500–1000 mL	0.825	0.281	0.003	2.283	1.317–3.959
			>1500 mL	<500 mL	1.567	0.376	0.000	4.791	2.295–10.005
		b	>1500 mL	1000–1500 mL	0.734	0.337	0.029	2.083	1.076–4.032
			>1500 mL	500–1000 mL	0.701	0.322	0.030	2.015	1.071–3.792
			>1500 mL	<500 mL	1.364	0.419	0.001	3.911	1.721–8.887
		c	>1500 mL	1000–1500 mL	0.586	0.354	0.098	1.798	0.897–3.601
			>1500 mL	500–1000 mL	0.557	0.341	0.102	1.745	0.895–3.403
			>1500 mL	<500 mL	1.131	0.444	0.011	3.097	1.298–7.389
The MCI group and the AD group (female)									
	Tea consumption								
		a	None	Green tea	−1.133	0.305	0.000	0.322	0.177–0.585
		b	None	Green tea	−0.970	0.333	0.004	0.379	0.197–0.729
		d	None	Green tea	−0.845	0.404	0.037	0.430	0.195–0.948
	Pure milk consumption								
		a	NO	YES	−0.936	0.253	0.000	0.392	0.239–0.644
		b	NO	YES	−0.892	0.286	0.002	0.410	0.234–0.718
		e	NO	YES	−0.818	0.330	0.013	0.441	0.231–0.843

Notes: a, basic model, no adjustment; b, adjusted for education, age, dieting to lose weight, diarrhea, allergy history, pro-cognitive drugs use; c, adjusted for all variables in model b + long-term drinking water, tea consumption, tea drinking frequency, pure milk consumption, yogurt consumption, APOE genotype; d, adjusted for all variables in model b + daily water consumption, long-term drinking water, coffee consumption, pure milk consumption, yogurt consumption, APOE genotype; e, adjusted for all variables in model b + daily water consumption, long-term drinking water, tea consumption, tea drinking frequency, coffee consumption, yogurt consumption, APOE genotype; MCI, mild cognitive impairment; AD, Alzheimer’s disease; B, Coefficient for the constant; SE, Standard error around the coefficient for the constant; *p*, *p*-value; OR, Exp (B); 95%CI, Confidence interval for the odds ratio with its upper and lower limits.

**Table 4:** Multivariate analysis of tea consumption and tea drinking frequency.

Group	Categorical Variable	Model	Reference Group		B	SE	*p*	OR	95%CI
The objective cognitive unimpaired group and the objective cognitive impairment group (male)									
	Tea consumption								
		a	None	Green tea	−1.157	0.270	0.000	0.314	0.185–0.533
		b	None	Green tea	−1.223	0.307	0.000	0.294	0.161–0.537
		c	None	Green tea	−1.068	0.323	0.001	0.344	0.182–0.647
	Tea drinking frequency								
		a	Rarely	≥3 cups/day	−1.003	0.308	0.001	0.367	0.201–0.671
			Rarely	1–2 cups/day	−0.870	0.338	0.010	0.419	0.216–0.812
			Rarely	<1 cups/day	−1.106	0.431	0.010	0.331	0.141–0.770
		b	Rarely	≥3 cups/day	−1.216	0.361	0.001	0.296	0.146–0.602
			Rarely	1–2 cups/day	−0.985	0.384	0.010	0.374	0.176–0.792
			Rarely	<1 cups/day	−1.405	0.512	0.006	0.245	0.090–0.669
		c	Rarely	≥3 cups/day	−1.005	0.381	0.008	0.336	0.174–0.772
			Rarely	1–2 cups/day	−0.816	0.403	0.043	0.442	0.201–0.974
			Rarely	<1 cups/day	−1.343	0.527	0.011	0.261	0.093–0.733

Notes: a, basic model, no adjustment. b, adjusted for education, age, marriage, allergic history, pro-cognitive drugs use. c, adjusted for all variables in model b + daily water consumption, coffee consumption, pure milk consumption, yogurt consumption, APOE genotype. B, Coefficient for the constant; SE, Standard error around the coefficient for the constant; *p*, *p*-value; OR, Exp (B); 95%CI, Confidence interval for the odds ratio with its upper and lower limits.

**Table 5:** Comparison of plasma biomarkers scales in pure milk and tea consumption.

Groups	Biomarkers (pg/mL) ^a^						
PET (+)							
	Pure milk consumption				Tea consumption		
		YES	NO	*p* ^b^	Green tea	None	*p* ^b^
	N	77	49		47	71	
	Aβ42	9.8 (3.7)	8.8 (3.7)	0.132	8.5 (4.0)	9.9 (3.5)	0.053
	Aβ40	199.7 (64.8)	198.8 (69.4)	0.941	186.0 (68.8)	209.2 (63.3)	0.066
	Aβ42/40	0.050 (0.014)	0.047 (0.017)	0.270	0.049 (0.017)	0.048 (0.014)	0.693
	t-Tau	2.5 (0.9)	2.6 (1.0)	0.332	2.4 (1.0)	2.6 (0.9)	0.553
	p-Tau-181	2.6 (1.9)	3.2 (1.5)	0.042	2.3 (1.6)	3.2 (1.8)	0.014
	NfL	20.0 (10.3)	19.4 (8.5)	0.740	18.1 (7.1)	20.5 (9.4)	0.144
PET (−)							
	Pure milk consumption				Tea consumption		
		YES	NO	*p* ^b^	Green tea	None	*p* ^b^
	N	87	54		69	65	
	Aβ42	10.8 (3.8)	10.5 (3.9)	0.571	10.6 (3.5)	10.9 (4.2)	0.663
	Aβ40	199.0 (57.8)	194.0 (71.2)	0.654	199.3 (60.4)	196.5 (67.3)	0.798
	Aβ42/40	0.056 (0.016)	0.055 (0.012)	0.776	0.055 (0.014)	0.056 (0.015)	0.625
	t-Tau	2.5 (1.2)	2.6 (1.3)	0.523	2.6 (1.3)	2.6 (1.3)	0.940
	p-Tau-181	1.8 (1.0)	1.7 (0.9)	0.498	1.8 (0.9)	1.7 (1.0)	0.850
	NfL	15.4 (9.6)	13.2 (4.2)	0.064	15.2 (9.2)	14.4 (6.7)	0.572

Notes: ^a^ Except Aβ42/40 (a ratio), the units of each plasma biomarker are pg/mL. ^b^ Continuous variables were analyzed using independent sample *t*-test. Aβ, amyloid-β; t-Tau, plasma total tau; p-Tau-181, plasma tau phosphorylated at threonine 181; NfL, neurofilament light chain.

**Table 6:** Comparison of plasma biomarkers in coffee and daily water consumption.

Groups	Biomarkers (pg/mL) ^a^								
PET (+)									
		Coffee consumption			Daily water consumption				
		YES	NO	*p* ^b^	>1500 mL	1000–1500 mL	500–1000 mL	<500 mL	*p* ^c^
	N	31	110		19	38	54	11	
	Aβ42	9.7 (3.8)	9.3 (3.6)	0.533	9.1 (3.6)	9.5 (3.7)	9.6 (3.8)	9.0 (3.6)	0.950
	Aβ40	201.8 (64.3)	198.4 (67.4)	0.798	176.4 (64.5)	208.7 (61.5)	203.4 (69.3)	190.8 (65.8)	0.334
	Aβ42/40	0.050 (0.017)	0.048 (0.015)	0.411	0.055 (0.015)	0.047 (0.017)	0.048 (0.014)	0.050 (0.014)	0.262
	t-Tau	2.5 (1.2)	2.5 (0.9)	0.972	2.6 (1.1)	2.4 (0.7)	2.6 (1.0)	2.8 (0.9)	0.700
	p-Tau-181	2.5 (1.9)	3.0 (1.7)	0.198	2.4 (2.1)	2.5 (1.3)	3.2 (1.9)	3.2 (1.8)	0.180
	NfL	20.4 (11.1)	19.5 (9.0)	0.628	15.6 (7.2)	19.3 (10.3)	21.7 (10.4)	20.1 (6.1)	0.134
PET (−)									
		Coffee consumption			Daily water consumption				
		YES	NO	*p* ^b^	>1500 mL	1000–1500 mL	500–1000 mL	<500 mL	*p* ^c^
	N	31	110		33	52	48	6	
	Aβ42	9.9 (3.8)	10.9 (3.8)	0.209	9.9 (4.4)	10.4 (3.8)	11.5 (3.7)	11.9 (2.5)	0.246
	Aβ40	196.5 (72.7)	197.3 (60.3)	0.953	179.4 (75.7)	200.2 (55.2)	207.8 (66.1)	202.5 (45.8)	0.277
	Aβ42/40	0.053 (0.015)	0.056 (0.014)	0.280	0.057 (0.013)	0.053 (0.015)	0.056 (0.015)	0.059 (0.009)	0.543
	t-Tau	2.6 (1.2)	2.5 (1.3)	0.891	2.8 (1.6)	2.5 (1.2)	2.4 (1.0)	2.8 (1.4)	0.525
	p-Tau-181	1.6 (0.9)	1.8 (1.0)	0.368	1.7 (1.0)	1.8 (1.0)	1.7 (0.9)	1.6 (0.6)	0.930
	NfL	15.4 (11.3)	14.3 (6.9)	0.527	16.0 (10.4)	14.5 (7.3)	14.2 (7.3)	14.5 (2.6)	0.783

Notes: ^a^ Except Aβ42/40 (a ratio), the units of each plasma biomarker are pg/mL. ^b^ Independent sample *t*-test. ^c^ One-way analysis of variance (ANOVA). Aβ, amyloid-β; t-Tau, plasma total tau; p-Tau-181, plasma tau phosphorylated at threonine 181; NfL, neurofilament light chain.

## Data Availability

The data presented in this study are available on request from the corresponding author. The data are not publicly available due to confidentiality issues.
